# Supplementary figures and images for: Usability and acceptability of oral fluid- and blood-based hepatitis C virus self-testing among the general population and men who have sex with men in Malaysia
Source: PLOS Glob Public Health. 2024 Jan 3;4(1):e0001770. doi: 10.1371/journal.pgph.0001770 (PMC10763960; doi:10.1371/journal.pgph.0001770)

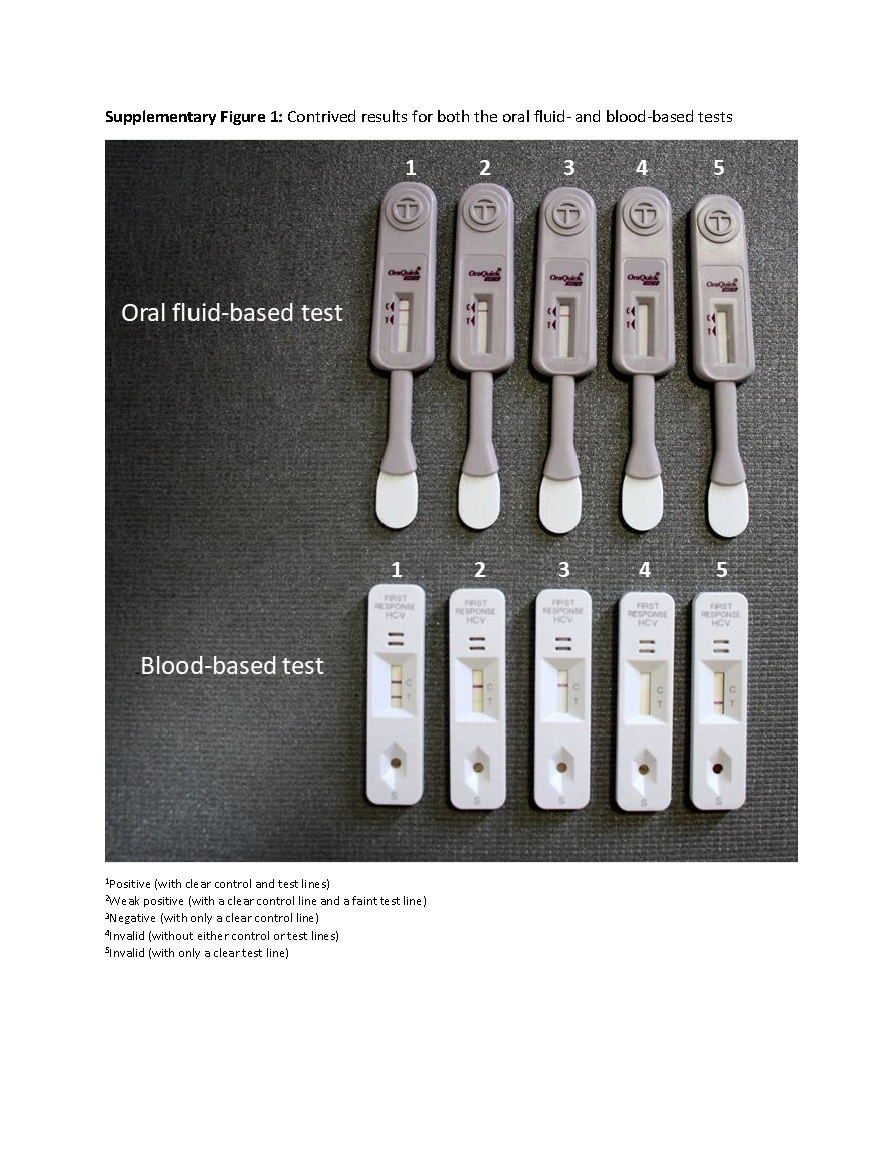

Supplement: S1 Fig — 1Positive (with clear control and test lines); 2Weak positive (with a clear control line and a faint test line); 3Negative (with only a clear control line); 4Invalid (without either control or test lines); 5Invalid (with only a clear test line). (TIF) [file pgph.0001770.s001.tif]
