# Supplementary material for: Usability and acceptability of oral fluid- and blood-based hepatitis C virus self-testing among the general population and men who have sex with men in Malaysia
Source: PLOS Glob Public Health. 2024 Jan 3;4(1):e0001770. doi: 10.1371/journal.pgph.0001770 (PMC10763960; doi:10.1371/journal.pgph.0001770)
Supplement: S1 Table — (DOCX) [file pgph.0001770.s002.docx]

S1 Table**.** **Assessment of inter-reader and inter-operator agreement in test result interpretation.**

| **Participant interpretation** | **Inter-reader concordance**  **(re-read by trained staff)** | | | | **Inter-operator concordance**  **(re-tested by trained staff)** | | | |
| --- | --- | --- | --- | --- | --- | --- | --- | --- |
|  | **Positive** | **Negative** | **Invalid** | **Total** | **Positive** | **Negative** | **Invalid** | **Total** |
| **Oral fluid-based test in GP (n = 100)** | | | | | | | | |
| Positive | 0 | 1 | 0 | 1 | 0 | 1 | 0 | 1 |
| Negative | 0 | 90 | 0 | 90 | 0 | 90 | 0 | 90 |
| Invalid | 0 | 2 | 7 | 9 | 0 | 8 | 1 | 9 |
| Total | 0 | 93 | 7 | 100 | 0 | 99 | 1 | 100 |
| Invalid rate (%) | 7.0 | | | | 1.0 | | | |
| Concordance (%) | 97.0 | | | | 91.0 | | | |
| Gwet’s AC1 coefficient | 0.76 | | | | 0.89 | | | |
| **Oral fluid-based test in MSM (n = 100)** | | | | | | | | |
| Positive | 0 | 0 | 0 | 0 | 0 | 0 | 0 | 0 |
| Negative | 0 | 97 | 1 | 98 | 0 | 98 | 0 | 98 |
| Invalid | 0 | 0 | 2 | 2 | 0 | 2 | 0 | 2 |
| Total | 0 | 97 | 3 | 100 | 0 | 100 | 0 | 100 |
| Invalid rate (%) | 3.0 | | | | 0.0 | | | |
| Concordance (%) | 99.0 | | | | 98.0 | | | |
| Gwet’s AC1 coefficient | 0.98 | | | | 0.99 | | | |
| **Blood-based test in GP (n = 100)** | | | | | | | | |
| Positive | 0 | 0 | 0 | 0 | 0 | 0 | 0 | 0 |
| Negative | 0 | 96 | 1 | 97 | 0 | 97 | 0 | 97 |
| Invalid | 0 | 0 | 3 | 3 | 0 | 3 | 0 | 3 |
| Total | 0 | 96 | 4 | 100 | 0 | 100 | 0 | 100 |
| Invalid rate (%) | 4.0 | | | | 0.0 | | | |
| Concordance (%) | 99.0 | | | | 97.0 | | | |
| Gwet’s AC1 coefficient | 0.98 | | | | 0.99 | | | |
| **Blood-based test in MSM (n = 100)** | | | | | | | | |
| Positive | 0 | 1 | 0 | 1 | 0 | 1 | 0 | 1 |
| Negative | 0 | 96 | 0 | 96 | 0 | 96 | 0 | 96 |
| Invalid | 0 | 1 | 2 | 3 | 0 | 3 | 0 | 3 |
| Total | 0 | 98 | 2 | 100 | 0 | 100 | 0 | 100 |
| Invalid rate (%) | 2.0 | | | | 0.0 | | | |
| Concordance (%) | 98.0 | | | | 96.0 | | | |
| Gwet’s AC1 coefficient | 0.60 | | | | 0.99 | | | |

GP, general population; MSM, men who have sex with men.
