# Supplementary material for: Usability and acceptability of oral fluid- and blood-based hepatitis C virus self-testing among the general population and men who have sex with men in Malaysia
Source: PLOS Glob Public Health. 2024 Jan 3;4(1):e0001770. doi: 10.1371/journal.pgph.0001770 (PMC10763960; doi:10.1371/journal.pgph.0001770)
Supplement: Attachment — Submitted filename: Response to Reviewers_Usability of HCVST in Malaysia.docx [file pgph.0001770.s003.docx]

Dear Dr. Andrew D. Kerkhoff,

Thank you for your and the reviewers’ comments and the opportunity to refine our manuscript entitled “Usability and acceptability of oral fluid- and blood-based hepatitis C virus self-testing among the general population and men who have sex with men in Malaysia”, for your consideration for publication in PLoS Global Public Health. We have addressed the comments below in blue font and in the uploaded manuscript document marked up. Please also find uploaded a clean version of the revised manuscript.

Journal Requirements:

1. We do not publish any copyright or trademark symbols that usually accompany proprietary names, eg ©, ®, ™ (e.g. next to drug or reagent names). Please remove all instances of trademark/copyright symbols throughout the text, including ® on page 7.

**Response:** Thank you for the comment. We have removed all instances of trademark/copyright symbols on page 7. It now reads as “All participants performed HCVST with two kits: OraQuick (oral fluid-based test kit; manufactured by OraSure Technologies, United States of America) and First Response (blood-based test kit; manufactured by Premier Medical Corporation, India). OraQuick (sensitivity: 98.1%; specificity: 99.6%) was already prequalified by the WHO for professional use at the time of this study, whereas First Response (sensitivity: 100%; specificity: 100%) was still under the review of the WHO for professional use.”

Comments to the Author

5. Review Comments to the Author

Reviewer #1: Excellent study, which is very timely as we aim to scale up options for HCV testing and surveillance. Unlike for HIVST where the market was shaped mostly around Oral Based tests, this study placed equal precedence on Oral and Blood which for me is an important factor. HCVST has the potential to close the ever widening knowledge gap amongst those affected with HCV, a treatable disease, and studies such as this are vitally important.

**Response:** Thank you very much for this positive feedback.

Reviewer #2: This is an interesting and timely article on HCV self-testing usability among MSM and the general population in Malaysia. It will make an important contribution to the literature and the authors are commended on great work. I especially enjoyed the insightful comments about groups developing capability to perform RDTs with repeated use, and the thorough exploration of HCVST usability. I have some concerns about the reporting of the findings and outline major revisions which will strengthen this paper for publication.

**Response:** Thank you very much for this positive feedback and the suggestions which we have responded to accordingly below.

Major

My most major concern is around how the figures for correct interpretation of positive results are reported. Currently the authors divide these up into ‘positive’ and ‘weak positive’ with only minimal discussion of the differences between these, and no discussion of the clinical implications of a weak positive. As all positive results (whether faint or strong) are indeed positive results and should be interpreted thus, it isn’t appropriate to only report these separately. Rather, all should be aggregated into one row and reported in the results. The authors may then also wish to report them separately in table 5. This is especially important given that participants often did not recognise ‘weak’ positive results (as high as 50% of the time for blood based tests in the general population).

A related point, I would suggest the authors expand with more detail on the implications of these issues with correctly interpreting tests results. Currently it received little attention despite being a critical issue to resolve before implementation.

**Response:** Thank you for the comments. We have expanded the implications of these issues by revising the following paragraphs in Results and Discussion respectively: Line 255 – “Participants were generally able to read positive, weak positive, negative, and invalid contrived results for both the oral fluid- and blood-based tests (Table 5). Clinically, all positive results (whether strong or weak) should be interpreted as positive.”; and Line 363 - “We observed some difficulties in interpreting weak positive results with a faint test line indicating to a need for additional guidance on how to interpret such results. Again, these difficulties may have arisen as participants in Malaysia were not yet sensitized to the use of RDTs and hence, such problems would reduce over time.”

The authors compare HCVST to HIVST a few places in the article. One critical point in this is missing which is that HCV antibody tests will remain positive after a person has cleared HCV, either through treatment or spontaneously during acute infection. That means that these tests are not necessarily as widely useful, especially in treatment experienced populations and key populations with repeated exposures. This issue also has really important implications for intervention design.

**Response:** Thank you for the comment. We have stated this important consideration in lines 60 to 63: “It is important to note that with HCVST, as it is an anti-HCV antibody test, a positive result may not indicate active viremia [15]. This differs from HIVST and requires supportive education such that test performers understand how to interpret what a positive HCVST may mean.”

Minor

Line 51 – authors state 9.4 million HCV-infected individuals benefited from testing and treatment. Two issues here, 1) authors should use person centred language (e.g. people with HCV) and 2) mention if the 9.4 million is a global number (if it is?)

**Response:** Thank you for the comment, We have revised and clarified the statement: “Although globally 9.4 million people with HCV benefited from simplified testing procedures and received DAA-based treatment between 2015 and 2019 [4], the suboptimal uptake of facility-based testing, largely due to the poor accessibility of primary care, concerns around stigma, and competing priorities [5, 6], has limited the expansion of treatment.”

Line 60 – the systematic review referencevd has been superseded by the following articles:

Witzel TC, Eshun-Wilson I, Jamil MS, Tilouche N, Figueroa C, Johnson CC, Reid D, Baggaley R, Siegfried N, Burns FM, Rodger AJ. Comparing the effects of HIV self-testing to standard HIV testing for key populations: a systematic review and meta-analysis. BMC medicine. 2020 Dec;18(1):1-3.

Eshun-Wilson I, Jamil MS, Witzel TC, Glidded DV, Johnson C, Le Trouneau N, Ford N, McGee K, Kemp C, Baral S, Schwartz S. A systematic review and network meta-analyses to assess the effectiveness of human immunodeficiency virus (HIV) self-testing distribution strategies. Clinical Infectious Diseases. 2021 Aug 15;73(4):e1018-28.

Jamil MS, Eshun-Wilson I, Witzel TC, Siegfried N, Figueroa C, Chitembo L, Msimanga-Radebe B, Pasha MS, Hatzold K, Corbett E, Barr-DiChiara M. Examining the effects of HIV self-testing compared to standard HIV testing services in the general population: A systematic review and meta-analysis. EClinicalMedicine. 2021 Aug 1;38:100991.

**Response:** Thank you for the comment. We have updated the reference accordingly to include the above three articles: “It has been shown to substantially enhance HIV testing uptake, especially among men who have sex with men (MSM) [11-14].”

Line 64 – oddly phrased. Suggest starting this sentence with something about how it is critical to generate local evidence on acceptability, values and preferences to inform HCVST implementation.

**Response:** Thank you for the comment. We have revised the paragraph to clarify the importance of generating local evidence: “It is critical that for countries keen to introduce HCVST into their testing policy, local evidence should be generated for acceptability, values and preferences to inform HCVST implementation and the WHO’s recommendations should be adapted accordingly to the national and local context.”

Line 72 – explain what FIND is

**Response:** Thank you for the comment. We have explained what FIND is in the revised paragraph: “Despite initial successes in the expansion of DAA-based treatment [17, 18], a large majority of those infected remain undiagnosed and the MOH continues to collaborate with FIND, a non-profit diagnostics initiative, to explore options increasing access to HCV screening.”

Line 111 – worth mentioning whether the tests were inactivated (I believe they were not)

**Response:** Thank you for the comment. We clarify that the tests used by participants to assess usability of participants to complete all steps in the testing process were active tests and that the tests used for the contrived results read were premade test cassettes and not active tests. The contrived results portion of the study was done to mitigate the chance that there were no positive results among the participants, so contrived tests were used to understand participants’ ability to interpret positive results. To clarify this, we have also revised the following lines in Methods>>HCV self-testing kits and Methods>>Study procedures respectively: line 118 - “Both tests were active.”; line 145 - “Next, participants were required to read contrived test results from premade test cassettes which included a mix of “positive”, “negative”, and “invalid” results (S1 Fig).”

Line 124 – how were people assigned to groups

**Response:** Thank you for the comment. We have revised the paragraph to clarify this: “To reduce usability bias, every other participant at each study site performed the oral fluid-based test first, while the remainder used the blood-based test first.”

Line 167 – reflect somewhere on why such a high proportion of the group with living with HIV. Was one of the study sites an HIV treatment centre?

**Response:** Thank you for the comment. Indeed, all three study sites are public primary care centres which also run special clinics for sexually transmitted diseases and provide free HIV treatment for citizens. We have revised the following paragraph in Methods>>Study design and setting lines 93-94 to reflect this: “Apart from providing general medical care, these three centres also run special clinics for sexually transmitted diseases (STDs), where approximately 70% of patients are MSM. These centres also provide free HIV treatment for Malaysian citizens.”

Line 327 – Language a bit confusing, worth clarifying

**Response:** Thank you for the comment. We have revised the paragraph to clarify: “Additionally, participants might have overlooked to keep the time and read results within the stipulated time period as they could be in a hurry to complete the study procedures.”

Line 366 – This issue could also be about domestic privacy, as is seen in a lot of settings with HIVST

**Response:** Thank you for the comment. We have revised the paragraph to include this possibility: “Only one-fifth of participants in our study preferred to perform HCVST by themselves at home. This may be due to lack of domestic privacy as observed with HIVST. This may also imply that participants lacked confidence to perform the test alone by themselves at the time of the study, despite the written and pictorial instructions provided.”

We thank you for taking the time to consider our revised article and we look forward to receiving your feedback.

Yours sincerely,

Elena Ivanova Reipold, Deputy Director for Technology Innovations

FIND

Campus Biotech, Chemin des Mines 9, 1202 Geneva, Switzerland

Email: elena.ivanova@finddx.org
