# Supplementary material for: Usability and acceptability of oral fluid- and blood-based hepatitis C virus self-testing among the general population and men who have sex with men in Malaysia
Source: PLOS Glob Public Health. 2024 Jan 3;4(1):e0001770. doi: 10.1371/journal.pgph.0001770 (PMC10763960; doi:10.1371/journal.pgph.0001770)
Supplement: Attachment — Submitted filename: Response to Reviewers_Usability of HCVST in Malaysia.docx [file pgph.0001770.s004.docx]

Dear Dr. Andrew D. Kerkhoff,

Thank you for your and the reviewers’ comments and the opportunity to refine our manuscript entitled “Usability and acceptability of oral fluid- and blood-based hepatitis C virus self-testing among the general population and men who have sex with men in Malaysia”, for your consideration for publication in PLoS Global Public Health. We have addressed the comments below (responses in blue font) and in the uploaded manuscript document marked up. Please also find uploaded a clean version of the revised manuscript.

**Journal Requirements:**

Please review your reference list to ensure that it is complete and correct. If you have cited papers that have been retracted, please include the rationale for doing so in the manuscript text, or remove these references and replace them with relevant current references. Any changes to the reference list should be mentioned in the rebuttal letter that accompanies your revised manuscript. If you need to cite a retracted article, indicate the article’s retracted status in the References list and also include a citation and full reference for the retraction notice.

**Response:** Thank you for the comment. We have reviewed our reference list, made changes and have ensured that it is now complete and correct.

**Additional Editor Comments (if provided):**

Thank you for your revised submission. In addition to addressing the additional reviewer comments, please also address the minor, stylistic points below, which are largely focused on improving the readability of the manuscript.

Minor:

- Line 32: Would highlight in the abstract that weak positive results were difficult to read as this is an important finding. For example… “including that of contrived results, although there was difficulty interpreting weak positive results.

**Response:** Thank you for the comment. We have added in the abstract Line 33 that “However, the majority of the participants were still able to obtain and interpret results correctly, including that of contrived results, although there was substantial difficulty interpreting weak positive results.”

- Remove Sentence at lines 81-82 as it does not substantially add to the rationale for the study.

**Response:** Thank you for the comment. We have removed the sentence “HCV self-testing would also be accepted by the general population in Rwanda and people who inject drugs (PWID) in Kyrgyzstan who had no actual experience of self-testing.”

- Line 175: Add “the” before majority.

**Response:** Thank you for the comment. We have added accordingly.

- Line 179: Add “compared to MSM” at the end of this sentence.

**Response:** Thank you for the comment. We have added accordingly

- Lines 198- 204: please separate each error by commas rather than a semi-colon.

**Response:** Thank you for the comment. We have separated each error by commas accordingly.

- Line 224: given the large amount of text that follows this subheader, consider adding specificity to this subheader and including an additional subheader at line 257 for improved clarity and readability.

**Response:** Thank you for the comment. We have now added specificity to the subheader by revising it to “Result interpretation after conducting HCVST) and have also included an additional subheader “Contrived result interpretation” at line 257 (now line 258) for improved clarity and readability.

- Line 263: Add “a” - Weak positives with (a) faint test line.

**Response:** Thank you for the comment. We have revised accordingly.

- Line 340: add “the” - … was skipping (the) hand washing step…

**Response:** Thank you for the comment. We have revised accordingly.

- Line 363: remove “to” - … indicating a need for…

**Response:** Thank you for the comment. We have revised accordingly.

- Line 373: it appears that a reference is missing.

**Response:** Thank you for the comment. The missing reference [12] is now added.

Table 1. Please round any p-values greater than 0.05 to two decimal points only. Further do not bold “significant values.” Also change “latest” to “Most recent” for HCV and HIV test results for improved clarity.

**Response:** Thank you for the comment. We have revised accordingly.

Table 2. Please round any p-values greater than 0.05 to two decimal points only. For consistency, add a row that corresponds to the “number of participants who made at least one error in pre-testing-steps.” For readability, it would be helpful to indent each of the rows that correspond to the above sub-header. This would help to more clearly separate each usability testing subsection. It would also help to not bold the cumulative measures and to also indent them under the corresponding header. Perhaps these could be italicized instead.

**Response:** Thank you for the comment. We have revised accordingly.

Table 3. Similar comments as Table 2 with regard to formatting considerations.

**Response:** Thank you for the comment. We have revised accordingly.

Table 4. Given the large number of tables included, I would consider making this a supplementary table given the key aspects are well summarized in the results section.

**Response:** Thank you for the comment. We have revised accordingly and have now made this table into supplementary table 1.

Table 5. Please round any p-values greater than 0.05 to two decimal points only. Consider adding a brief footer that gives a quick summary of what a ‘contrived” test is for readers less familiar with usability testing and this term.

**Response:** Thank you for the comment. We have revised accordingly, this is now table 4.

Table 6. Please round any p-values greater than 0.05 to two decimal points only.

**Response:** Thank you for the comment. We have revised accordingly, this is now table 5.

**Reviewer's Responses to Questions**

**Comments to the Author**

Reviewer #2: Thank you for the opportunity to review this revised manuscript. The majority of my comments have been addressed and I feel this article is clearer. I remain enthusiastic about this publication and think it will make an important contribution to the literature.

I maintain substantial concern around the discussion of weak positives, and it feels as though important findings are being downplayed. The difficulty in interpreting weak positives is a critical finding which problematises HCVST and must be address for successful implementation. Given that half of participants were not able to correctly interpret faint positive blood-based HCVSTs (and nearly half oral fluid ones), the proposed revisions are not objective (or reflective of reality).

The authors state: “Participants were generally able to read positive, weak positive, negative, and invalid contrived results for both the oral fluid- and blood-based tests (Table 5). Clinically, all positive results (whether strong or weak) should be interpreted as positive.”; and Line 363 - “We observed some difficulties in interpreting weak positive results with a faint test line indicating to a need for additional guidance on how to interpret such results. Again, these difficulties may have arisen as participants in Malaysia were not yet sensitized to the use of RDTs and hence, such problems would reduce over time.” The participants were indeed not generally able to read weak positive blood-based results and the difficulties should not be characterised as ‘some’ but rather as ‘substantial’. It’s helpful to highlight the need for further advice, what other intervention components could be included? Finally, the changes also need to be reflected in the abstract so that those skimming the article are aware from the outset.

**Response:** Thank you for the comments.

We have now revised and stated in the abstract Line 33 that “However, the majority of the participants were still able to obtain and interpret results correctly, including that of contrived results, although there was substantial difficulty interpreting weak positive results.”; in the main text Lines 258-259 that “Participants were generally able to read positive, negative, and invalid contrived results for both the oral fluid- and blood-based tests (Table 4).”; in the main text Lines 267-269 that “However, weak positive results with a faint test line and a clear control were least accurately interpreted by the participants (51.3% for blood-based HCVST and 64% for oral fluid-based HCVST).”; and in the main text Line 367 that “We observed substantial difficulties in interpreting weak positive results with a faint test line, indicating a need for additional guidance on how to interpret such results.”

We have also expanded the implications of these issues in the main text Lines 369-373 that “Supportive materials such as manufacturer’s IFU, video instructions and flyers would need to highlight and emphasize on how to interpret weak positive results; moreover, these materials should also provide information ensuring that if people with risk factors are not able to understand or interpret such weak positive results, they would have sufficient support to access facility-based testing by healthcare workers.”

We thank you for taking the time to consider our revised article and we look forward to receiving your feedback.

Yours sincerely,

Elena Ivanova Reipold

Deputy Director for Technology Innovations

FIND

Campus Biotech, Chemin des Mines 9, 1202 Geneva, Switzerland

Email: elena.ivanova@finddx.org
